# A causative role for periarticular skeletal muscle weakness in the progression of joint damage and pain in OA

**DOI:** 10.1038/s41598-023-46599-7

**Published:** 2023-12-04

**Authors:** Ju-Ryoung Kim, Thi Hong Nhung Pham, Wan-Uk Kim, Hyun Ah Kim

**Affiliations:** 1 Division of Rheumatology, Department of Internal Medicine, Hallym University Sacred Heart Hospital, 896, Pyungchon, Anyang, Kyunggi 14068 Korea; 2 Institute for Skeletal Aging, Hallym University, Gangwon-Do, 24252 Korea; 3 Division of Rheumatology, Department of Internal Medicine, School of Medicine, The Catholic University of Korea, Seoul, 06591 Korea; 4 Center for Intergrative Rheumatoid Transcriptomics and Dynamics, The Catholic University of Korea, Seoul, 06591 Korea

**Keywords:** Acute inflammation, Chronic inflammation

## Abstract

Although osteoarthritis (OA) is regarded as a disease of the articular cartilage, recent research has demonstrated alterations in periarticular muscles that surround the affected joint. Here, we investigated changes in periarticular muscle during the progression of OA, as well as the cause-and-effect relationship between muscle weakness and OA, in a mouse model of OA by destabilization of the medial meniscus (DMM). Pathological phenotypes in the periarticular muscles were assessed in the early and late stages of OA by DMM. OA pathology and pain behavior in the mice after DMM induction were examined in response to periarticular muscle weakness induced by multiple rounds of barium chloride (BaCl_2_) injections. The examinations were also performed in myostatin knockout mice with strengthened muscle phenotypes by muscle hypertrophy. Morphological alterations in the tibialis anterior (TA) and quadriceps muscles in DMM mice included variations in muscle-fiber size, aberrant extracellular matrix (ECM) deposition, inflammatory cell infiltration, and decreased muscle mass. Periarticular muscle fibers isolated from DMM mice showed reductions in the number of satellite cells and myogenic capacity of primary myoblast, as well as proliferation. DMM + muscle injury mice also showed exacerbated joint degeneration compared to the DMM vehicles. Myostatin knockout mice were characterized by attenuated OA and the complete abrogation of pain behavior after DMM. Our results suggest an association between muscle weakness and OA progression and pain.

## Introduction

Osteoarthritis (OA) is the most prevalent joint disease causing disability, and the knee is the most commonly affected joint^1^. Although OA is regarded as a disease of the articular cartilage, recent research has demonstrated whole-joint pathology, including synovial inflammation, subchondral bone sclerosis, osteophyte formation, and changes in periarticular muscles that surround the affected joint^2^. There is also increasing evidence that the consequences of knee OA such as pain and joint instability are associated with decreased lower limb muscle strength^2^. Quadriceps muscle mass and strength determine lower extremity functions, and the progressive loss of both has been demonstrated in patients with OA^3^. Additionally, greater quadriceps strength protects against cartilage loss^4^. Studies of the molecular regulation of periarticular muscle loss and knee OA have revealed increased levels of inflammatory mediators including monocyte chemoattractant protein-1 (MCP-1) in the muscles of patients with knee OA^5^. These findings suggest that muscle weakness or atrophy accelerates the inflammatory process in the joint, leading to exacerbation of cartilage degeneration. Conversely, quadriceps muscle-focused strength training effectively relieves pain, and improves physical function as well as structural changes in OA patients^6^. It is unclear whether the change in periarticular muscle is the cause or result of disease progression, however.

Animal models of OA exhibit muscle mass loss similar to the loss in human OA patients. In an animal model involving the induction of OA by anterior cruciate ligament transection (ACLT), gastrocnemius muscle wasting coincided with elevated expression of interleukin (IL)-1β within the muscle^7^. ACLT-induced OA also promoted tibialis anterior (TA) muscle wasting, in a manner associated with inflammatory signs^8^. The induction of muscle mass loss via botulinum type-A toxin injections in rabbits led to cartilage degradation within 4 weeks after injury, suggesting that muscle weakness is a direct cause of degenerative joint disease^9^.

In this study, we investigated the mechanism of muscle atrophy in a widely used mouse model of knee OA, achieved by destabilization of the medial meniscus (DMM). The cause-and-effect relationship between knee OA progression and periarticular muscle weakness was examined in mice with muscle injury and muscle hypertrophy. The relationship between pain response and muscle change was also explored in those mice.

## Methods

### Animals, OA induction and muscle injury

All methods were carried out in accordance with the relevant guidelines and regulations. Animal experiments were performed in accordance with protocols approved by the Care and Use of Laboratory Animals Ethical Committee of the National Veterinary Research & Quarantine Service of South Korea and the Hallym Medical Center Institutional Animal Care and Use Committee (HMC 2017-1-0831-25). All methods are reported in accordance with ARRIVE guidelines. Specific pathogen-free 8-week-old C57BL/6 male mice were purchased from Dooyeol Biotech (Seoul, Korea) and been acclimated for 2 weeks before surgery. The mice cages were maintained in temperature-controlled (23 ± 2.0 °C) and humidity-controlled (50 ± 10%) environment with 12 h cycle of light and dark. OA was induced in the right knee joint of 10-week-old mice by surgical DMM^10^. Sham-operated mice were used as controls. Detailed procedures are provided in Supplementary Data. TA and quadriceps muscles, as well as knee joints, were collected from each designated time points until 12 weeks after DMM. Muscle injury was induced by injecting BaCl_2_ (1.2%, 45 μl, Sigma-Aldrich, 217,565) into the quadriceps muscle four times at 10-day intervals. Ten-week-old male C57BL/6 mice were divided into four experimental groups: sham vehicle, sham + muscle injury, DMM vehicle, and DMM + muscle injury as provided in Supplementary Fig. 4A and B. Sham-operated or DMM vehicle mice were injected with phosphate-buffered saline. Quadriceps muscles were isolated for analysis at 3 weeks after the last injection.

Myostatin knockout (Mstn^−/−^) mice were generously provided by Professor Yun-Sil Lee (Seoul National University) and DMM was performed to induce OA in 10-week-old male mice.

### Histology and immunohistochemistry

The TA and quadriceps muscles were embedded in tragacanth (Sigma-Aldrich, 9000-65-1) on a slice of cork and directly frozen in liquid nitrogen-cooled isopentane. Samples including the rectus femoris (RF) part of quadriceps were cryosectioned at a thickness of 10 μm for histological evaluation. Hematoxylin and eosin (H&E) staining was performed as previously described^11^. Picrosirius red staining was performed using the protocol and reagents of the Picrosirius Red staining kit (American MasterTech, KTPSRPT) after tissue sections had been fixed in Bouin’s solution (Sigma-Aldrich, HT01032) for 30 min at 56 °C. Knee joints were fixed with 4% paraformaldehyde, decalcified in 0.5 M ethylenediaminetetraacetic acid (pH 7.4), embedded in paraffin, and sectioned coronally through the entire depth of the joint. The Sects. (5-μm thickness) were stained with safranin-O (0.1%) and fast green. Each section was scored by two blinded observers using the Osteoarthritis Research Society International (OARSI) scoring system^12^. Subchondral bone sclerosis, osteophytes, and synovitis were also scored. For immunohistochemistry, joint sections were treated with hyaluronidase (0.1 units) for 30 min at 37 °C, permeabilized with 0.2% Triton X-100 for 5 min, and blocked with 5% goat serum in phosphate-buffered saline. The following primary antibodies were used: anti-type II collagen (Abcam, ab34712, dilution 1:200), anti-aggrecan (Millipore, AB1031, dilution 1:200), and anti-matrix metalloproteinase (MMP)-13 (Abcam, ab39012, dilution 1:200). The secondary antibody was goat anti-rabbit IgG (Santa Cruz, sc-2004, dilution 1:200). Positive results were visualized using 3,3’-diaminobenzidine (DAB) substrate staining (Vector Laboratories, SK-4100). All histology specimens were imaged on a Nikon ECLIPSE microscope equipped with a digital camera (Nikon DS-Fi2).

### Statistical analysis

GraphPad Prism 8 software was used for statistical analysis. The normality of the data was determined using the Shapiro–Wilk test. Parametric t- tests and Mann–Whitney U test was used for comparisons between two groups with normally and non-normally distributed data, respectively. For comparisons of > 2 conditions, one-way analysis of variance (ANOVA) or the Kruskal–Wallis test was used (depending on data distribution normality), followed by Tukey’s multiple comparisons test or Dunn’s multiple comparisons test, respectively. Pain behavior data did not follow normal distribution and was analyzed using the Kruskal–Wallis test with Dunn’s multiple comparisons test at each time point. All data are expressed as means ± standard errors of the mean (SEMs).

Detailed materials and methods are provided as in Supplementary Data.

## Results

### Knee OA contributes to morphological alterations in periarticular skeletal muscle

**Figure 1:**
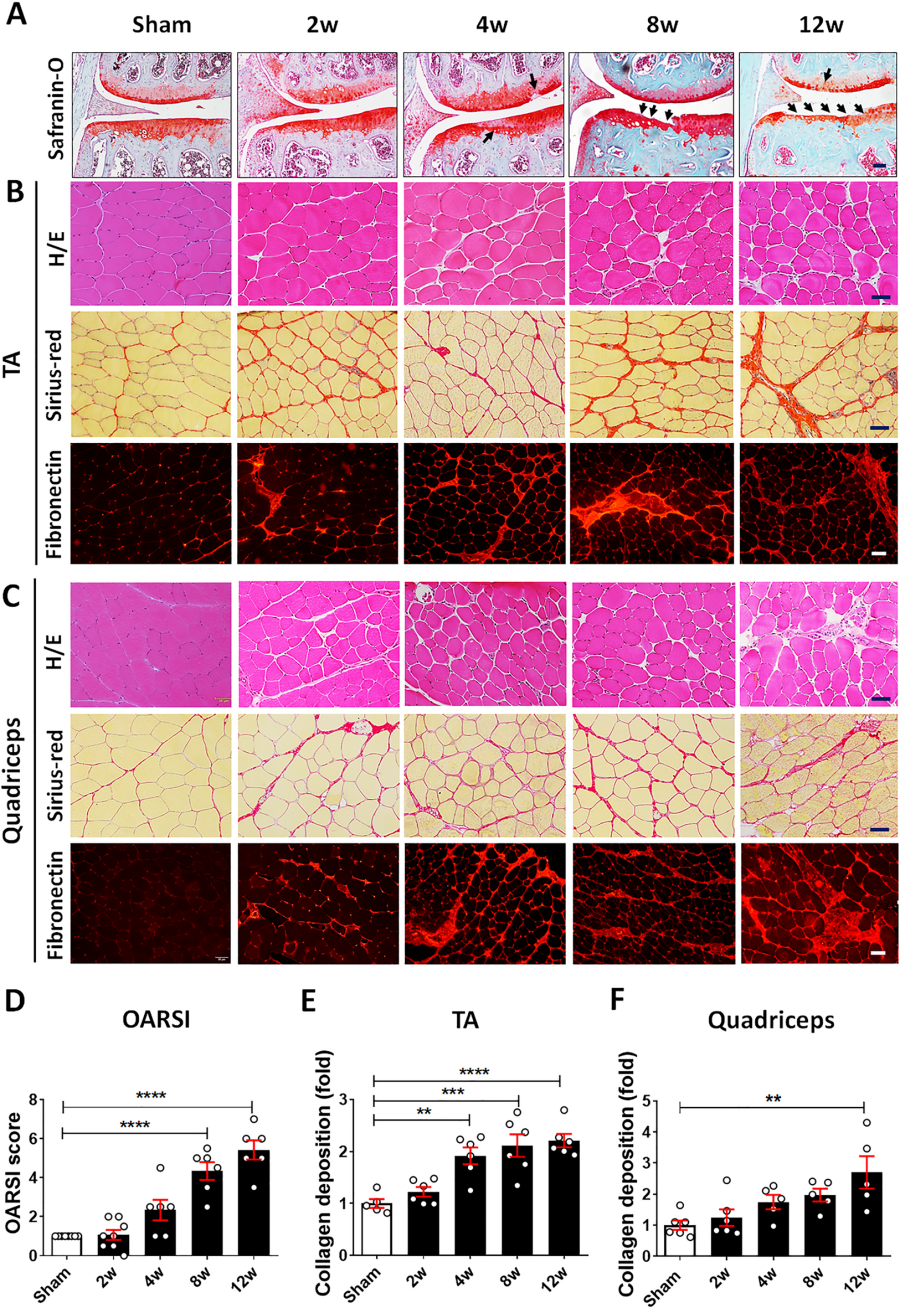
Morphological changes in periarticular muscle in a mouse model of osteoarthritis (OA) resulting from destabilization of the medial meniscus (DMM). (**A**) Representative images of the knee joints of sham-operated mice and DMM mice at 2, 4, 8, and 12 weeks after DMM or sham surgery. Histopathological changes were visualized by safranin-O/fast green staining. Arrows show cartilage degeneration. Scale bars = 50 μm. (**B**) and (**C**) Representative cross-sections of the tibialis anterior (TA) (**B**) and quadriceps (**C**) muscles stained with H&E (upper panels), picrosirius red (middle panels), and immunofluorescence for fibronectin (lower panels) at the indicated time points after DMM. Scale bars = 50 μm. (**D**) The extent of cartilage destruction was evaluated using the Osteoarthritis Research Society International (OARSI) grading system (n = 6–8, three sections/mouse). (**E**) and (**F**) Quantification of picrosirius red staining of the TA (**E**) and quadriceps (**F**) muscles at the indicated time points after DMM (n = 5–6, six images/mouse section). Data are shown as means ± SEMs based on comparisons with sham-operated mice. **P < 0.01, ***P < 0.001, ****P < 0.0001 according to one-way ANOVA with Tukey’s multiple comparisons test.

Muscle and joint tissues were obtained at 2 and 4 weeks and at 8 and 12 weeks after DMM for early and late-stage OA, respectively. Proteoglycan loss from the cartilage matrix of the tibial plateau and the femoral condyle was apparent at 4 weeks (Fig. 1A). Cartilage destruction into deep zones of the tibial plateau was observed at 8 weeks; severe cartilage erosion and denudation had developed by 12 weeks (Fig. 1A). Beginning 8 weeks after DMM, the OARSI score (reflecting cartilage damage severity) was significantly higher in DMM mice than in sham-operated mice (Fig. 1D). Assessments of morphological changes in the TA and quadriceps muscles revealed uniform orientation and similar size of muscle fibers in both muscles in the sham-operated mice. However, variations in muscle-fiber size, round muscle fibers, and extracellular matrix remodeling, with enhanced endomysial and perimysial connective tissue surrounding the muscle fibers, became apparent at 2 weeks after DMM and then exhibited increasing severity at late stages (Fig. 1B,C). To investigate changes in extracellular matrix organization, we investigated collagen deposition examined by picrosirius red staining and fibronectin expression. In the DMM group, the amounts of collagen in the TA and quadriceps muscles increased in parallel with OA progression (Fig. 1B,C). At 4, 8 and 12 weeks after DMM, the increases in collagen accumulation in the TA and quadriceps muscles, respectively, were significant compared to sham (Fig. 1E,F). In addition, abnormal expression of fibronectin was also detected in both muscles during OA development indicating pathophysiological response of ECM.

### OA progression is accompanied by muscle atrophy

**Figure 2:**
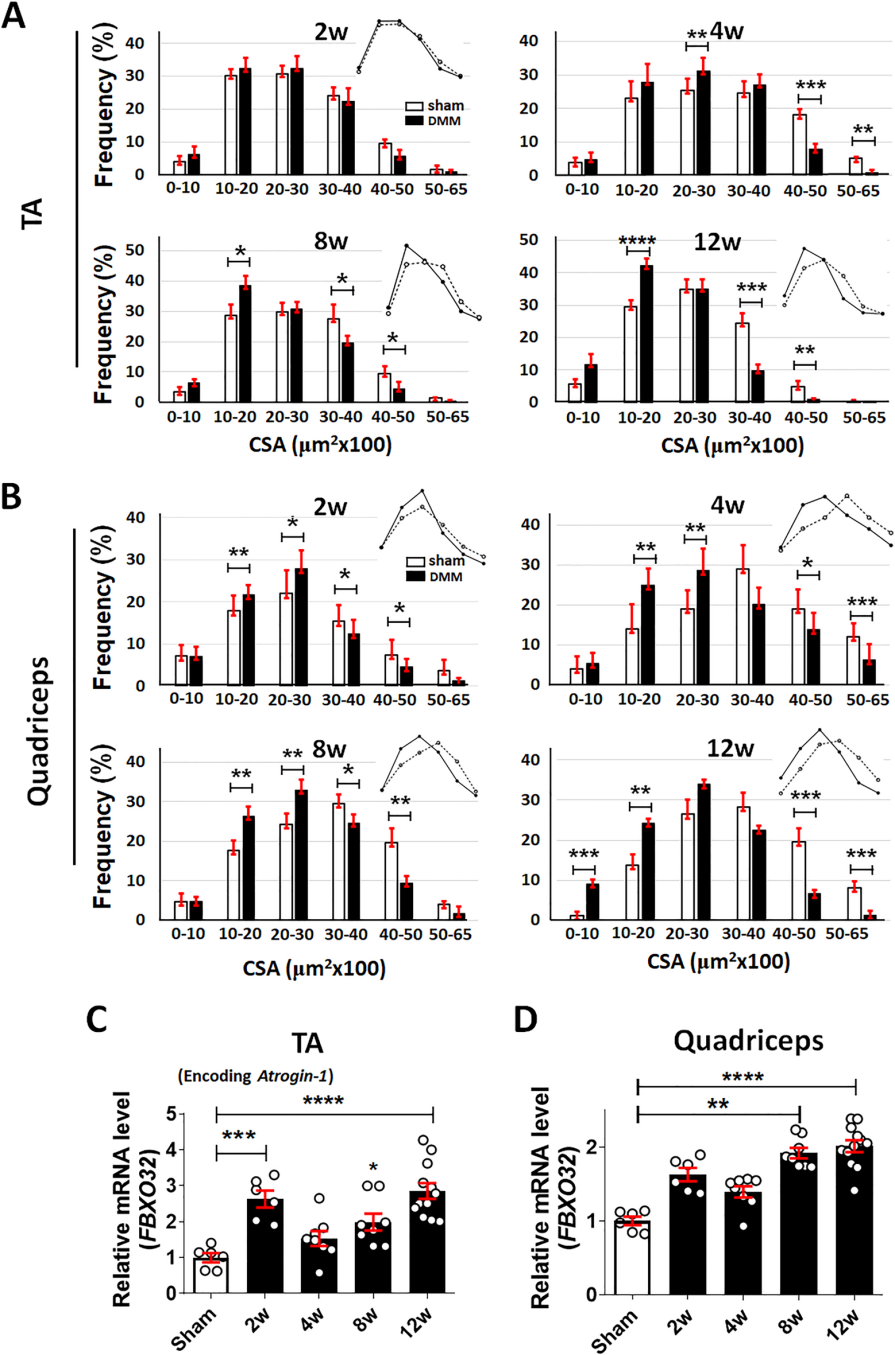
Muscle atrophy accompanies OA progression. (**A**) and (**B**), Distributions of the fiber cross-sectional area (μm^2^) of the TA (**A**) and quadriceps (**B**) muscles at 2, 4, 8, and 12 weeks postoperatively. Muscle-fiber size was measured on H&E-stained muscle sections. For each mouse sample, at least six images of muscle sections from different microscopic fields were examined (n = 5 for the sham group and n = 5–7 for the DMM group). (**C**) and (**D**), Expression levels of FBXO32 mRNA in the TA (**C**) and quadriceps (**D**) muscles of the sham-operated (n = 6) and DMM (n = 6–12) groups, as determined by real-time qRT-PCR. Data are normalized to the expression of glyceraldehyde-3-phosphate dehydrogenase (GAPDH) mRNA and shown as means ± SEMs of duplicate experiments from 3 to 6 mice. *P < 0.05, **P < 0.01, ***P < 0.001, ****P < 0.0001 according to unpaired two-tailed *t*-tests (**A**,**B**), one-way ANOVA with Tukey’s multiple comparisons test (**C**) and the Kruskal–Wallis test with Dunn’s multiple comparisons test (**D**).

The relationship between OA progression and periarticular muscle atrophy was examined by measuring the weights of the TA and quadriceps muscles in DMM-induced OA mice. DMM mice showed significant decrease, 10% and 11% in the ratio of TA muscle mass to body weight at 2 and 12 weeks and 16%, 16% and 17% in quadricep muscle at 4, 8 and 12 weeks compared to sham, respectively. (Supplementary Fig. 1). Muscle-fiber cross-sectional area was then measured to quantitatively evaluate muscle-fiber atrophy in the OA mice. Both the TA and quadriceps muscles exhibited a significant leftward shift in fiber size, with a higher frequency of small fibers during the OA development except for at 2 weeks of TA muscle (Fig. 2A,B). Because the degradation of skeletal muscle protein is partially regulated by the E3 ubiquitin proteasome pathway, the regulation of a key signaling gene in this pathway, atrogin-1, was examined. An increase in F-box protein 32 (FBXO32; encoding atrogin-1) expression, beginning at 2 weeks and persisting until 12 weeks after DMM, was observed in both the TA and quadriceps muscles (Fig. 2C,D). Taken together, these results showed that periarticular muscle atrophy begins at OA onset and persists until the late stage of the disease.

### Development of OA leads to periarticular muscle inflammation

**Figure 3:**
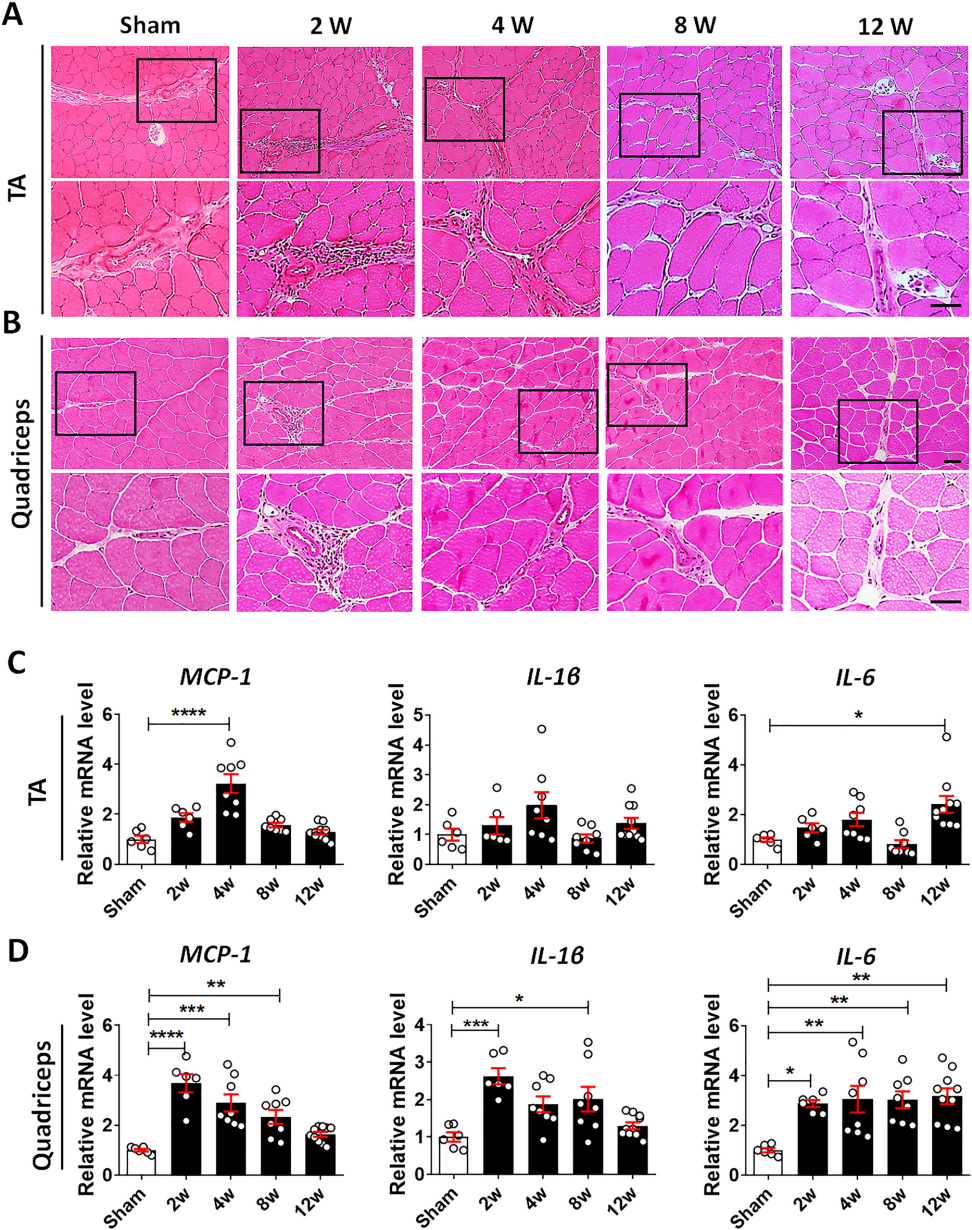
Inflammatory response in skeletal muscle after DMM. (**A**) and (**B**) Representative images from H&E-stained sections of the TA (**A**) and quadriceps (**B**) muscles at 2, 4, 8, and 12 weeks postoperatively. For each muscle, inflammatory cell infiltration (box) is shown as a magnified image in the lower panels. Scale bars = 50 μm. (**C**) and (**D**) Total RNA was extracted from the TA (**C**) and quadriceps (**D**) muscles at 2, 4, 8, and 12 weeks postoperatively; the levels of monocyte chemoattractant protein 1 (MCP-1), interleukin (IL)-1β, and IL-6 mRNA were analyzed using real-time qRT-PCR. Data are normalized to the expression of GAPDH mRNA and shown as means ± SEMs of duplicate experiments from 3–5 animals. *P < 0.05, **P < 0.01, ***P < 0.001, ****P < 0.0001 according to one-way ANOVA with Tukey’s multiple comparisons test for MCP-1 (TA, quadriceps), IL-1β, and IL-6 (quadriceps) and according to the Kruskal–Wallis test with Dunn’s multiple comparisons test for IL-1β and IL-6 (TA).

Next, we examined whether OA progression induces an inflammatory response in periarticular muscle. Hematoxylin and esosin (H/E) staining revealed that inflammatory cells were present in the TA and quadriceps muscles of DMM mice throughout the 12-week study period (Fig. 3A,B). Quantitative reverse transcription-polymerase chain reaction (qRT-PCR) was performed to determine whether the expression levels of inflammatory cytokines and chemokines were elevated during OA progression. In the quadriceps, the levels of monocyte chemoattractant protein 1 (MCP-1) significantly increased from 2 to 8 weeks, then decreased (Fig. 3C,D). IL-1β levels in the quadriceps were significantly upregulated at 2 and 8 weeks post DMM, whereas IL-6 levels significantly increased at all time points. The increase in inflammatory mediators was less conspicuous in TA than in quadriceps muscle. These findings indicate that knee OA progression induces a pathological inflammatory state in periarticular skeletal muscle that persists until the late stage of OA.

### OA development is associated with decreased myogenic commitment of satellite cells

**Figure 4:**
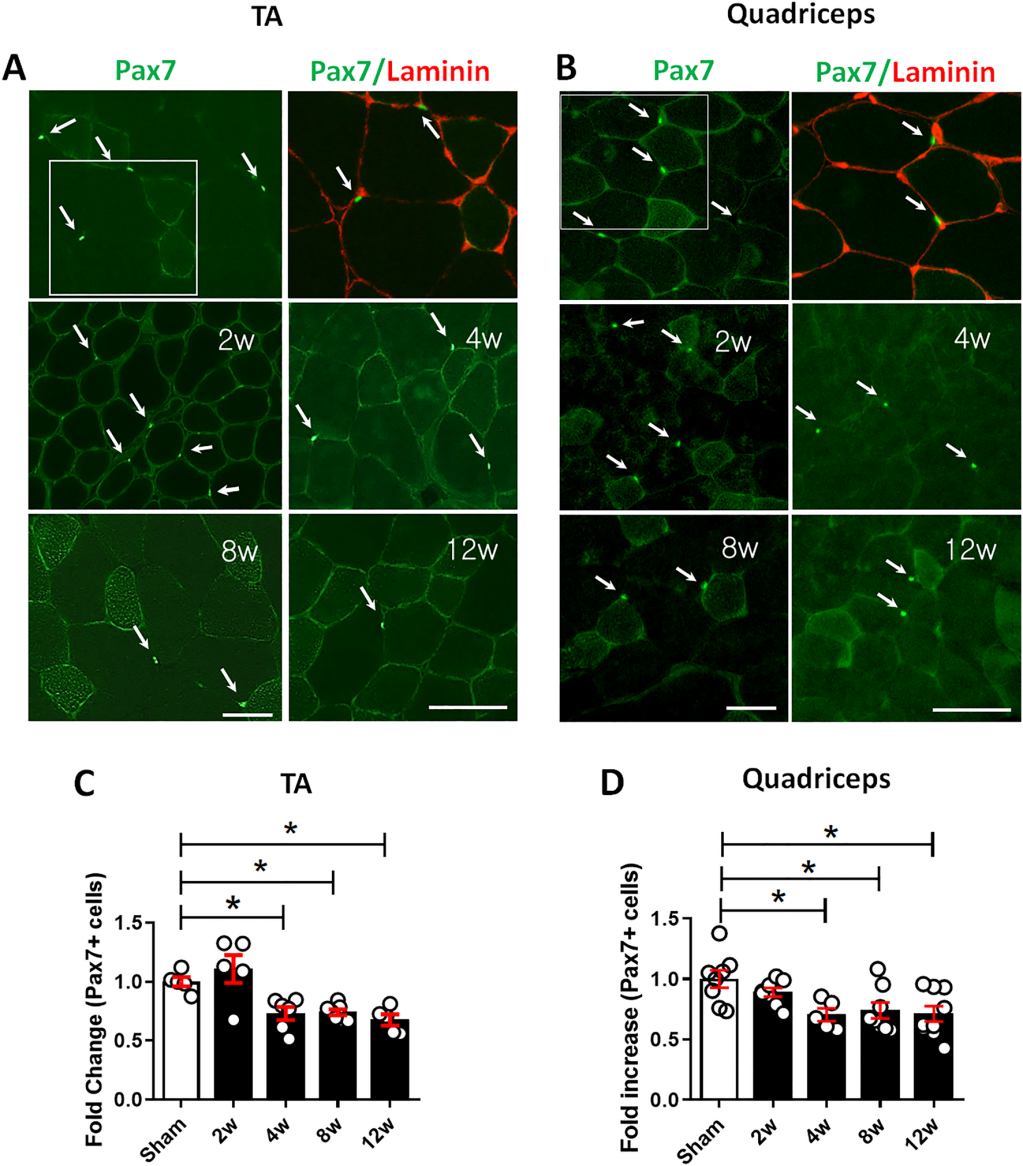
OA development is accompanied by a decrease in the number of pax7-positive satellite cells. (**A**) and (**B**) Representative immunofluorescence images of pax7 (green, arrows) and laminin (red) staining in sections of the TA (**A**) and quadriceps (**B**) muscles at 2, 4, 8, and 12 weeks after DMM or sham surgery. Scale bars = 50 μm. (**C**) and (**D**) Quantification of the number of pax7-positive satellite cells in sections of the TA (**C**) and quadriceps (**D**) muscles at 2, 4, 8, and 12 weeks after DMM or sham surgery (n = 5–9). Data are shown as means ± SEMs. *P < 0.05 according to one-way ANOVA with Tukey’s multiple comparisons test.

A decrease in satellite cells, precursors to skeletal muscle cells may explain the decreased regenerative potential of periarticular muscle in OA mice. Indeed, fewer Pax7, a satellite cell marker, expressions in the TA and quadriceps muscles were observed in DMM than in sham mice (Fig. 4A,B), reflecting significantly lower satellite cells from 4 to 12 weeks after DMM (Fig. 4C,D). To further investigate whether OA progression affects the myogenic capacity of myoblasts, primary myoblasts (i.e., myogenic precursor cells) were isolated from the TA and quadriceps muscles of sham and DMM mice at 4 and 12 weeks, then permitted to differentiate for 5 days. The presence of shorter myotubes with less fusion in cultures from DMM, than cultures from sham-operated mice, indicated a lower capacity for myogenic differentiation (Supplementary Fig. 2A). Furthermore, fewer MyHC-positive myotubes were observed in DMM compared with sham-operated mice, consistent with the lower fusion index in cells from DMM mice (Supplementary Fig. 2B,C). Five days after differentiation, the mRNA expression levels of myogenic markers (e.g., MyoD, myogenin, and desmin) were lower in cells obtained from DMM than in cells obtained from the respective sham mice (Supplementary Fig. 2D–F). Differences in 5-ethynyl-2'-deoxyuridine (Edu) staining revealed 40% and 23% reduction in proliferation rate at 4 and 12 weeks in primary myoblasts from DMM than sham-operated mice, respectively (Supplementary Fig. 3A).

### Multiple rounds of periarticular muscle injury induce severer muscle atrophy in OA induced mice

**Figure 5:**
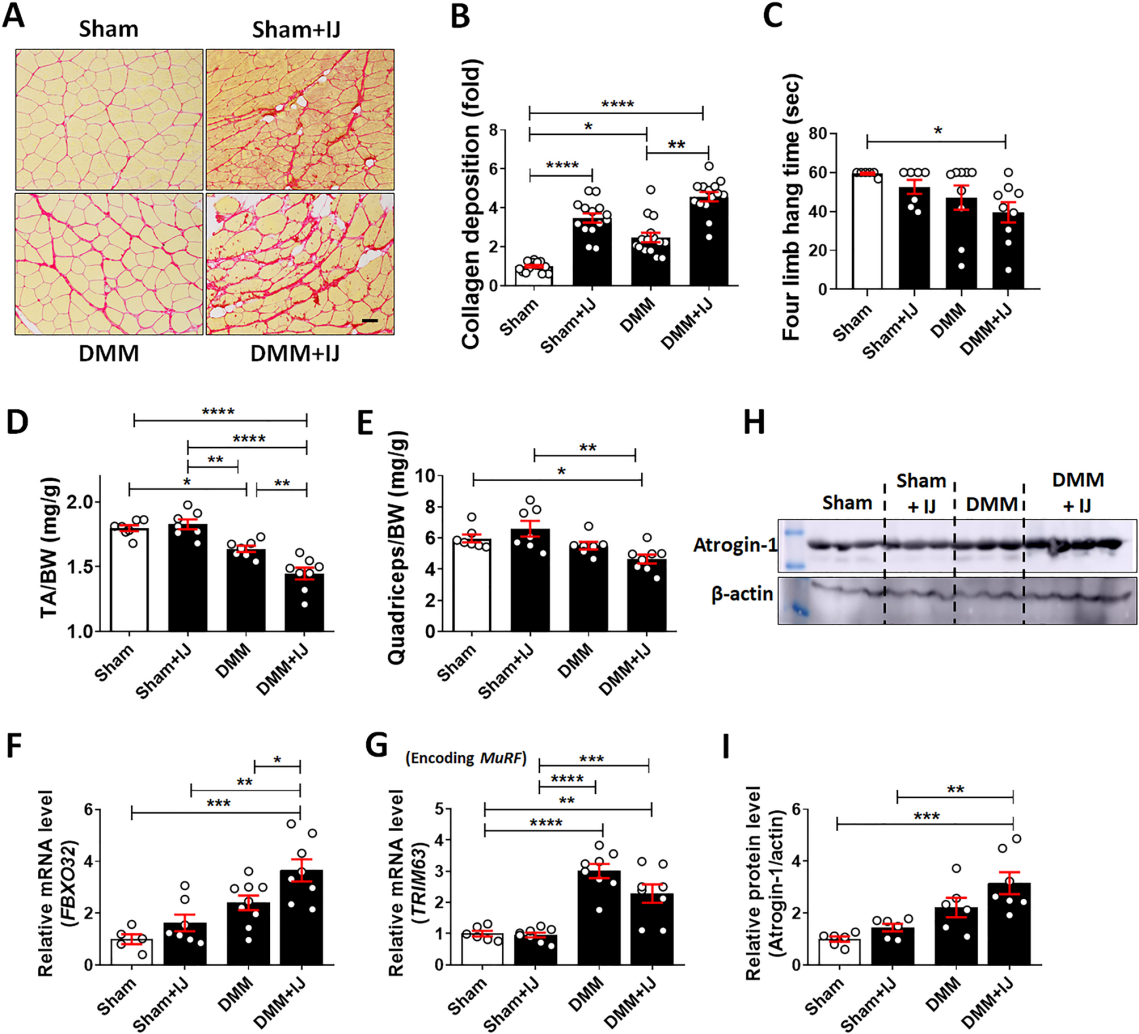
Induction of muscle weakness in the quadriceps muscles of DMM and sham-operated mice by four injections of BaCl_2_, separated by 10-day intervals. Sham and DMM vehicle groups were injected with phosphate-buffered saline. (**A**) Representative images of quadriceps muscles stained with picrosirius red. Scale bars = 50 μm. (**B**) Quantification of the collagen deposition by picrosirius red staining. For each mouse sample, at least six images of muscle sections from different microscopic fields were examined (n = 7). (**C**) Four limb hang measurement to assess muscle strength at 9 weeks after DMM (n = 6–9). (**D**) and (**E**) Relative TA (**D**) and quadriceps (**E**) muscle mass (n = 7–8) in the sham, sham + injury, DMM, and DMM + injury groups. (**F**) and (**G**) Expression levels of FBXO32 (**F**) TRIM63 (**G**) mRNA in the quadriceps muscles of mice from each of the four groups. Data are normalized to the expression of GAPDH mRNA and shown as means ± SEMs of duplicate experiments from 5 to 9 mice. (**H**) Representative western blot images of atrogin-1 in quadriceps muscles of mice from each of the four groups. Three independent experiments were performed. (**I**) Quantitative analysis of atrogin-1 protein levels. Values were normalized to the level of β-actin (n = 6), and data are shown as means ± SEMs. *P < 0.05, **P < 0.01, ***P < 0.001, ****P < 0.0001 according to one-way ANOVA with Tukey’s multiple comparisons test (**D**,**F**,**G**,**I**) or according to the Kruskal–Wallis test with Dunn’s multiple comparisons test (**B**,**C**,**E**).

We then investigated whether muscle weakness promotes OA progression in DMM mice. Ten-week-old male C57BL/6 mice were divided into four experimental groups: sham vehicle, sham + muscle injury, DMM vehicle, and DMM + muscle injury (Supplementary Fig. 4). Compared with sham vehicle mice, mice in the muscle injury groups showed robust endomysial ECM deposition and fatty replacement (Fig. 5A), as well as a 15% reduction in muscle strength measured by four limb-hanging test (Fig. 5C); these findings indicated pathophysiological changes of muscle and a decrease in muscle strength upon repeated muscle injury. DMM + injury mice exhibited more aberrant ECM and fatty deposition compared with sham + IJ or DMM mice (Fig. 5A,B). In addition, DMM and DMM + injury showed 21% and 33% decrease in muscle strength compared with sham mice, respectively (Fig. 5C). Muscle injury led to a further significant reduction in muscle mass in the DMM mice (Fig. 5D,E). Although the TA was not directly injured, its mass decreased to a greater extent in DMM + injury than in DMM vehicle mice, presumably because of the systemic inflammation induced by multiple rounds of injury. The expression levels of FBXO32 and TRIM63 genes were significantly higher in DMM mice than in sham-operated mice, and FBXO32 level was even higher in the DMM + injury group (Fig. 5F,G). The changes in atrogin-1 protein levels were consistent with changes in FBXO32 mRNA levels (Fig. 5H,I).

### Periarticular muscle weakness exacerbates OA development

**Figure 6:**
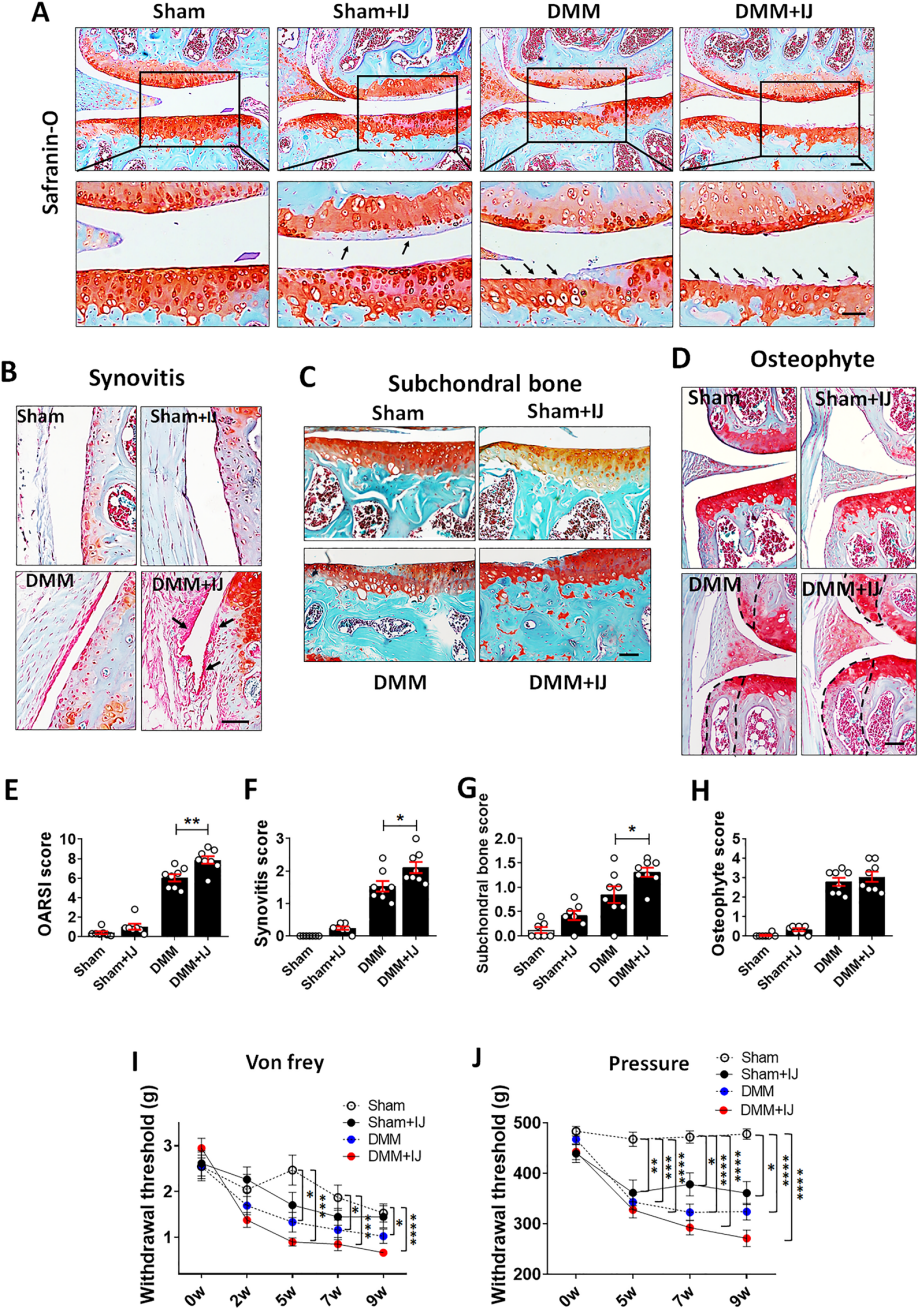
Exacerbation of OA development after the post-DMM induction of muscle weakness. (**A**) Cartilage degeneration was visualized by safranin-O/fast green staining at 9 weeks after DMM. Boxed areas are shown at higher magnification below. Note the reduced safranin-O staining and the clefts on the joint surface in the BaCl_2_-injected (sham + injury), DMM-vehicle-, and BaCl_2_-injected DMM (DMM + injury) groups. Arrows indicate areas of cartilage degeneration. Scale bars = 50 μm. (**B**) Representative images of the synovium in the knee joint of each group. Arrows indicate increased synovial proliferation. (**C**) Representative images of the subchondral bone in the knee joint of each group. (**D**) Representative images of osteophyte formation in the knee joint. Dashed lines in the tibia and femur indicate the newly formed osteophyte area. (**E**–**H**) The OARSI scores (**E**), synovitis scores (**F**), subchondral bone scores (**G**), and osteophyte formation scores (**H**) are shown for each group (n = 8). (**I**) Mechanical allodynia in the left hind paw of mice in each group (n = 8) was evaluated using von Frey filaments. (**J**) Withdrawal threshold, as measured using a pressure application measurement (PAM) force transducer at the left knee, of mice in each of the four groups (n = 7–8). Data are shown as means ± SEMs. *P < 0.05, **P < 0.01, ***P < 0.001, ****P < 0.0001 according to unpaired two-tailed *t*-tests in (**E**–**G**) (DMM vs. DMM + IJ). Kruskal–Wallis test with Dunn’s multiple comparisons test was applied for the comparison of withdrawal thresholds at different time points (**I**,**J**). In (**I**,**J**), P values were based on a comparison between sham mice.

Analysis of the effects of muscle weakness on joint degeneration revealed more severe cartilage erosion and denudation within the tibial plateau in DMM + injury mice than in DMM mice, with significantly higher OARSI score in DMM + injury mice (Fig. 6A,E). Although reduced proteoglycan staining of the femoral condyle was also observed in sham + injury group, the OARSI score was not significantly higher in sham + injury than in sham group (Fig. 6A,E). Additionally, greater synovial thickening and more intense synovitis scores were observed in DMM + injury than in DMM mice (Fig. 6B,F). In contrast, only a slight increase in synovitis was observed in the sham + injury group, compared with the sham vehicle group (Fig. 6B,F). Muscle injury led to a significant increase in subchondral bone sclerosis compared with DMM alone (Fig. 6C,G), but osteophyte formation did not differ between the two groups (Fig. 6D,H). Thus, our results indicate that periarticular muscle weakness exacerbates joint destruction. Joint pain in each group of mice was assessed using pain behavior test designed to determine whether muscle weakness also aggravates OA-associated pain. The results showed that DMM led to reduced withdrawal threshold measured with either von Frey filament and pressure algometer after 5 weeks, which persisted until 9 weeks postoperatively (Fig. 6I,J). The withdrawal threshold in DMM + injury was lower than DMM vehicle mice, albeit not significantly.

### Myostatin knockout mice exhibit reduced joint degeneration after DMM

**Figure 7:**
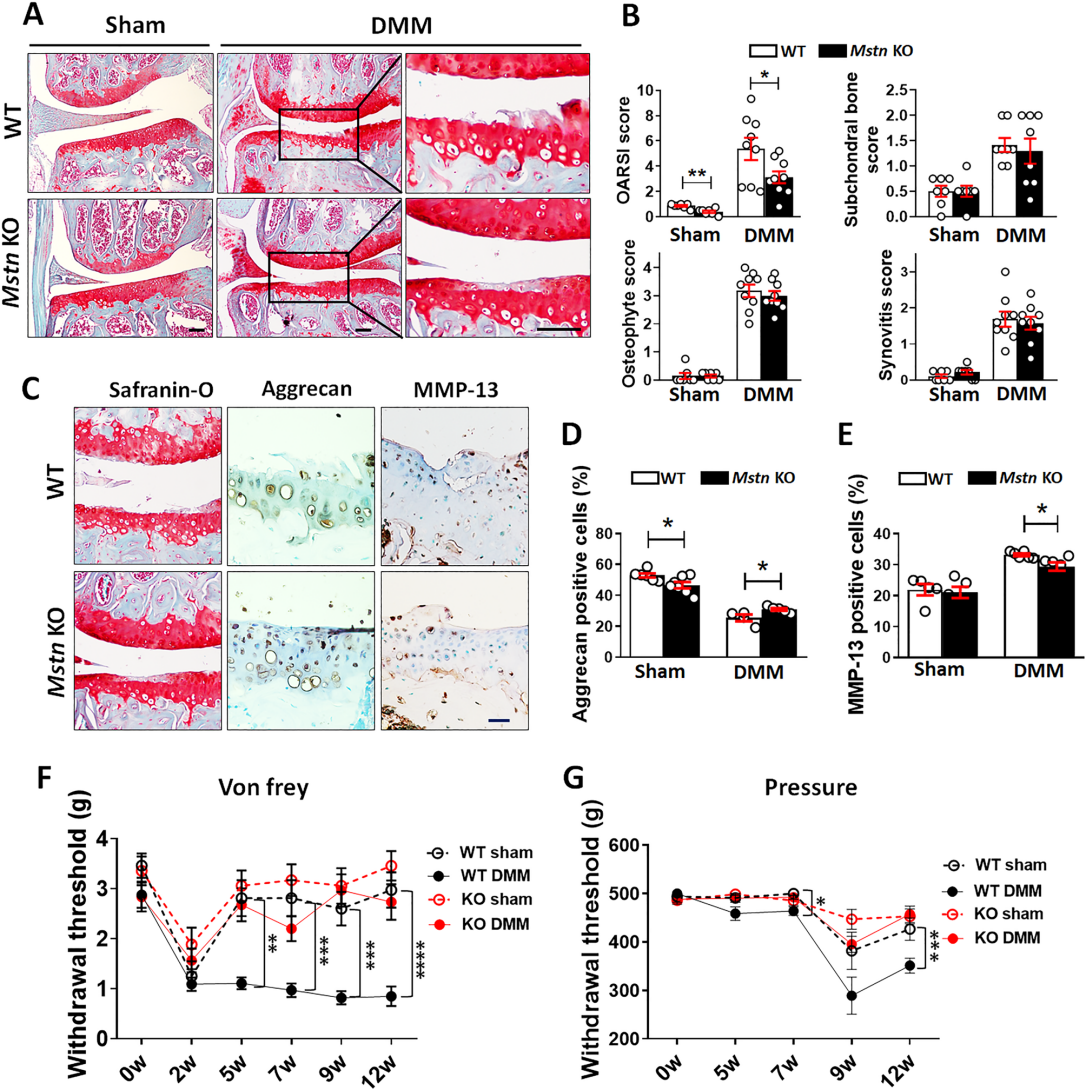
Attenuated joint degeneration in myostatin knockout (Mstn KO) mice after DMM. (**A**) Representative images of safranin-O/fast green stained sections of the knee joints of wild-type (WT) and Mstn KO mice after DMM. Boxed areas are shown at higher magnification in the right panel. Scale bars = 50 μm. (**B**) Quantitative analyses of the OARSI score, synovitis score, subchondral bone score, and osteophyte formation after DMM in Mstn KO and WT mice. (n = 7–9). (**C**) Representative images of immunohistochemical staining for aggrecan and MMP-13 in knee sections from WT and Mstn KO mice after DMM. Scale bars = 50 μm. (**D**) and (**E**) Quantification of aggrecan ((**D**), n = 4–7) and MMP-13 ((**E**), n = 5–6) staining. (**F**) and (**G**) Mechanical allodynia was evaluated using von Frey filaments ((**F**), n = 8–10), and the withdrawal threshold was measured using a pressure application measurement (PAM) device ((**G**), n = 4–6) after DMM. Data are shown as means ± SEMs. *P < 0.05, **P < 0.01, ***P < 0.001, ****P < 0.0001. Statistical analyses were conducted using unpaired two-tailed *t*-tests in (**B**) (OARSI, osteophyte, and DMM group synovitis score), (**D**), and (**E**); a two-tailed Mann–Whitney U test in (**B**) (subchondral bone sclerosis and sham group synovitis score). Kruskal–Wallis test with Dunn’s multiple comparisons test was applied for the comparison of withdrawal thresholds at different time points (**F**,**G**). In (**F**,**G**), P values were based on a comparison between WT sham mice.

Myostatin is a negative regulator of muscle growth. Previous reports showed that pharmacological inhibition of myostatin increases skeletal muscle mass and improved muscle strength^13–15^. Furthermore, myostatin protein expression was increased in the gastrocnemius muscle in ACLT induced OA animal model in other report^7^. Therefore, we examined whether muscle growth and enhanced muscle strength could protect against OA development in myostatin knockout (Mstn KO) mice after DMM surgery. We found no significant difference in cartilage thickness between 3- or 8-week-old Mstn KO mice and wild-type (WT) mice, except for slightly increased tibial plateau cartilage thickness in 3-week-old Mstn KO mice (Supplementary Fig. 5A,B). We also found no differences in the expression patterns of anabolic matrix genes (e.g., ACAN, Col2a1, and Sox9) between Mstn KO and WT mice, as determined by qRT-PCR (Supplementary Fig. 5C). The expression patterns of the respective proteins, evaluated by immunohistochemistry, supported this result (Supplementary Fig. 5D). Ten-week-old Mstn KO and WT mice were subjected to DMM surgery; their knee joints were collected 12 weeks later. Mstn KO mice did not show decrease in muscle mass or FBXO32 gene expression of TA and quadriceps contrary to WT mice after DMM (Supplementary Fig. 6A,B). After DMM, cartilage erosion at the medial tibial plateau was less severe in Mstn KO mice than in WT mice, with significantly lower OARSI score (Fig. 7A,B); however, there were no significant differences in synovitis, subchondral bone sclerosis, or osteophyte formation (Fig. 7B). An increase in aggrecan expression and a decrease in MMP expression were observed in Mstn KO mice compared to WT mice after DMM (Fig. 7C–E). Throughout the study period, Mstn KO mice completely did not exhibit reduced withdrawal threshold measured either both von Frey filament and pressure algometer after DMM suggesting the abrogation of OA-associated pain (Fig. 7F–G).

## Discussion

The present study investigated the relationship between knee OA progression and periarticular muscle weakness in a mouse DMM model. A reduction of periarticular muscle mass and the exacerbation of joint degeneration after muscle injury were observed in DMM mice. However, in Mstn KO mice with muscle hypertrophy, OA after DMM was less severe and the mice exhibited no signs indicative of pain.

Although periarticular muscle atrophy commonly accompanies human OA and is correlated with OA progression and pain, the pathologic role of muscle in OA and pain has been scarcely explored. Consistent with our findings, Cunha et al. observed that ACLT-induced OA in rats was accompanied by muscle atrophy and increased atrogin-1 expression in the peri-articular muscles^8^. Previous studies demonstrated involvement of local inflammation and inflammatory mediators in the induction of muscle atrophy^16–18^. We showed increases in inflammatory cell infiltration of the muscles and in the levels of inflammatory mediators such as MCP-1, IL-1β and IL-6 which were significantly evident in the quadriceps muscle within 2 weeks after DMM, suggesting that inflammatory signaling at the onset of OA induces pathological changes in muscles. Consistent with these results, a previous study showed both a higher level of IL-1β expression in the gastrocnemius muscle and a 10% reduction of myofiber cross-sectional area in an ACLT-induced model of knee OA^7^. Similarly, increases in MCP-1 and atrogin-1 expression were demonstrated in the vastus lateralis muscle of knee OA patients; the expression levels of both proteins were negatively correlated with muscle strength^5^.

An in-depth analysis of changes in periarticular muscle in DMM revealed excessive ECM accumulation including collagen and fibronectin, consistent with the findings of a study involving OA patients, where collagen deposition between muscle fibers was increased and exhibited a correlation with decreasing quadriceps strength^19^. There is evidence that satellite cell density is significantly lower in OA patients and that extracellular matrix accumulation is inversely correlated with satellite cell density^19^. Similarly, we observed a decline in satellite cell number in the TA and quadriceps muscles of DMM mice, in both the early and late stages of OA; satellite cell density was negatively correlated with aberrant ECM deposition. Additionally, the myogenic differentiation capacity and the proliferation of primary myoblasts, both of which are required for repairing muscle damage and promoting myofiber growth, were impaired after DMM. This pathophysiological changes in periarticular muscle during the OA progression indicating that muscle atrophy is a feature of OA.

Muscle weakness is a risk factor for OA progression, both in humans and in animal models^20–22^. Among the methods used to experimentally induce muscle injury, we chose repeated BaCl_2_ injection because it causes myofiber proteolysis, muscle fibrosis, and unresolved inflammation, which closely resemble the histological changes observed in periarticular muscle during OA progression after DMM. OA severity was significantly greater in DMM + injury mice than in DMM vehicle mice. Notably, articular cartilage destruction, subchondral bone sclerosis, and synovitis were significantly worse in DMM + injury mice than in DMM vehicle mice. Although, muscle injury tended to lead to higher pain sensitivity in DMM + injury mice than in DMM vehicle mice, it is unclear whether the increased pain behavior was an indirect effect of the exacerbation of joint destruction or a direct, intrinsic effect of muscle atrophy on pain hypersensitivity. Previous study showed that BaCl_2_ impairs chondrogenic differentiation through inhibition of potassium channel^23^. It is note that some sham animals injected with BaCl2 showed decrease in the proteoglycan content of cartilage, which may have resulted from either periarticular muscle atrophy or direct effect of BaCl2 transferred from the periarticular muscle to the cartilage. However, this result is consistent with previous reports of a significant relationship between muscle weakness and pain in OA patients^24, 25^.

Contemporary clinical guidelines for the management of knee OA recommend lower extremity strengthening exercises as primary non-pharmacological therapy. The mechanism by which exercise improves knee OA symptoms is unknown^26^; it may include increased muscle mass and strength or other, muscle-independent factors. The direct role of muscle mass regulation in the pathogenesis of OA has been investigated using Mstn KO mice because myostatin is a critical negative regulator of muscle growth^27^. Mstn KO mice develop muscle hypertrophy but lack a distinct cartilage phenotype compared with WT mice; these findings were based on assessments of femoral condyle and tibial plateau thicknesses, proteoglycan loss or erosion, and the expression patterns of anabolic matrix genes. However, upon induction of OA by DMM, we observed significant protection of articular cartilage destruction in Mstn KO mice than in WT mice. Moreover, DMM-induced OA pain sensitivity was completely abrogated in the Mstn KO mice. The phenomenon of exercise-induced hypoalgesia, in which physical activity leads to less musculoskeletal pain, has been attributed to the modulation of central nervous system excitability and the inhibition of psychological constructs associated with pain^28^. However, our results suggest that muscle-intrinsic factors also contribute to the attenuation of pain. Myokines are secreted muscle proteins that act as anti-inflammatory mediators, whose benefits as analgesic modulators received recent attention. For example, infusion of brain-derived neurotrophic factor (BDNF), a skeletal muscle myokine into the bilateral infralimbic cortices reportedly activated neuronal activities and alleviated the inflammatory pain induced with complete Freund’s adjuvant, while accelerating long-term recovery from pain^29, 30^. The production of insulin-like growth factor-1 in skeletal muscle is induced by exercise, and its expression was positively correlated with variation in the pain threshold during exercise in fibromyalgia^31^. The mediators of pain attenuation in Mstn KO OA mice merit further investigation.

Our study has limitations. Despite the decrease in muscle mass, it was difficult to prove that it leads to muscle weakness by our experimental method. Thus, the relationship between muscle strength and OA is speculative. Pain behavior tended to be more pronounced in the DMM + injury group compared to DMM, however, significance was not reached. The influence of muscle atrophy on pain may be small compared to that of DMM per se.

In summary, using DMM mice model of OA, we showed that OA leads to muscle weakness and inflammatory change. Manipulation of muscle mass by using BaCl_2_ or myostatin KO phenotype led to aggravation or amelioration of OA change, respectively, as well as pain behavior. Muscle strengthening offers a promising therapeutic approach to mitigate the development of OA and to protect against OA-associated pain.

### Supplementary Information

Supplementary Information.

## Data Availability

All processed data are available in the figures of the manuscript. All raw data that support the findings of this study are available from the corresponding authors upon request.
