# Supplementary material for: A causative role for periarticular skeletal muscle weakness in the progression of joint damage and pain in OA
Source: Sci Rep. 2023 Dec 4;13:21349. doi: 10.1038/s41598-023-46599-7 (PMC10696078; doi:10.1038/s41598-023-46599-7)
Supplement: Supplementary file 1 — Supplementary Information. [file 41598_2023_46599_MOESM1_ESM.docx]

**A causative role for periarticular skeletal muscle weakness in the progression of joint damage and pain in OA**

Ju-Ryoung Kim, PhD^1,2^, Thi Hong Nhung Pham^1,2^, Wan-Uk Kim^3,4^, Hyun Ah Kim, MD, PhD^1,2^*

^1^Division of Rheumatology, Department of Internal Medicine, Hallym University Sacred Heart Hospital, Kyunggi, 14068, Korea, ^2^Institute for Skeletal Aging, Hallym University, Gangwon-do 24252, Korea, ^3^Division of Rheumatology, Department of Internal Medicine, School of Medicine, The Catholic University of Korea, Seoul 06591, Korea, ^4^Center for Intergrative Rheumatoid Transcriptomics and Dynamics, School of Medicine, The Catholic University of Korea, Seoul 06591, Korea

***Correspondence to:**

Name: Hyun Ah Kim

Address: Division of Rheumatology, Department of Internal medicine, Hallym University Sacred Heart Hospital, 896, Pyungchon, Anyang, Kyunggi, 14068, Korea

Phone: 82-31-380-1826

Fax: 82-31-381-8812

E-mail: [kimha@hallym.ac.kr](mailto:kimha@hallym.ac.kr)

<https://orcid.org/0000-0002-9318-7446>

**Supplementary Methods**

**OA induction.**  DMM surgery of the right knee was performed in 10-week- old male C57BL/6 or Mstn KO mice^1^. Briefly, local anesthetic solution was prepared as follows. Zoletil 50 (a mixture of 1:1 tiletamine/zolazepam) powder was reconstructed at a concentration of 50 mg/mL by addition of 5 mL of autoclaved water and the stock mixture was diluted with Rompun (xylazine hydrochlorid, 23.32 mg/mL) and PBS pH 7.4 at 1:4:10 ratios. Mice were anaesthetized by intraperitoneal injection of an anesthetic solution (120 μl per mouse) subjected to surgery under a dissection microscope. The joint capsule medial to the patellar tendon was then incised with scissors, and the cranial meniscotibial ligament of the medial meniscus was transected. For sham surgery, the cranial meniscotibial ligament was exposed but the ligament was left intact. The joint capsule and skin were closed using 6-0 blue nylon surgical sutures. Mice were sacrificed at 12 weeks after surgery.

**Histologic evaluation using the Osteoarthritis Research Society International (OARSI), synovitis, subchondral bone, and osteophyte scoring systems.** To evaluate the extent of articular cartilage degeneration, a series of 5 µm-thick 3 sections taken at 200 μm intervals in the coronal plane were stained with Safranin-O (0.1%) and fast green, and hematoxylin. Each section was scored by two blinded observers using the OARSI, synovitis, subchondral bone, and osteophyte scoring systems. The OARSI scoring as follows^2^, 0, normal; 0.5, loss of Safranin-O with no structural lesions; 1, small fibrillations with roughened articular surface; 2, vertical clefts down to the layer immediately below the superficial layer and some loss of surface lamina; 3, vertical clefts/erosion to the calcified cartilage extending to < 25% of the articular surface; 4, vertical clefts/erosion to the calcified cartilage extending to 25–50% of the articular surface; 5, vertical clefts/erosion to the calcified cartilage extending to 50–75% of the articular surface; and 6, vertical clefts/erosion to the calcified cartilage extending to > 75% of the articular surface. The synovitis score was carried out according to the two synovial membrane features including synovial lining cell layer and inflammatory infiltrate), the ranking of changes being on a scale from none (0: one layer of lining cells and no inflammatory infiltrate), slight (1: 2-3 layers of lining cells and few mostly perivascular situated lymphocytes) and moderate (2: 4-5 layers of lining cells and numerous lymphocytes or plasma cells) to strong (3: the lining might be ulcerated, more than 5 layers and dense band-like inflammatory infiltrate) ^3^. The subchondral bone scoring as follows^4^, 0, less subchondral bone thickness than articular cartilage thickness in the load bearing region; 1, equal subchondral bone thickness to articular cartilage thickness in the load bearing region; 2, greater subchondral bone thickness to articular cartilage thickness in the load bearing region. The osteophyte formation scoring as follows^5^, 0, none; 1, formation of cartilage-like tissues; 2, increase of cartilaginous matrix; 3, endochondral ossification.

**Muscle-fiber cross-sectional area analysis.** Cross-sectional area was quantified on H&E muscle cross-sections. Fibers were manually outlined, then measured using ImageJ software (version 1.52a, NIH, USA). At least six images of muscle sections from different microscopic fields were examined in each mouse sample.

**Myoblast isolation and immunofluorescence.** TA and quadriceps muscles were collected, minced with scissors, and digested with collagenase and dispase as previously described^6^. Dissociated muscles were transferred to Falcon tubes and suspended in medium (20% fetal bovine serum/5% horse serum in Dulbecco’s modified Eagle medium), centrifuged, resuspended in the same medium, and filtered through a 70-μm sterilized cell strainer. The cells were first plated onto uncoated culture dishes for 30 min; subsequently, non-adherent cells were plated on 8-well chamber slides (Sigma-Aldrich, C7182) coated with Matrigel (Corning, 354277). When the cell were confluent in 70%), the cells were subjected to immunofluorescence analysis by incubation with primary antibody against Pax7 (DSHB, dilution 1:10) and laminin (Sigma, L9393, dilution 1:1000) for overnight at 4 ℃, followed by incubation with secondary antibody (Alexa 488 goat anti-mouse IgG_1_, Invitrogen, A21121 for pax7, and Alexa 546 goat anti-Rabbit IgG, Invitrogen, A11035 for laminin) for 30 min at room temperature. After 5 days of differentiation in differentiation medium (2% horse serum in Dulbecco’s modified Eagle medium), the cells were subjected to immunohistochemistry analysis by incubation with primary antibody against myosin heavy chain (MyHC) (DSHB, MF-20, dilution 1:200) for 1 h at room temperature, followed by incubation with secondary antibody (Alexa 488 goat anti-mouse IgG (H+L, Invitrogen, A11001) for 30 min at room temperature.

**Quantitative RT-PCR.** Total RNA was extracted and purified from TA and quadriceps muscles, cartilage tissue, or primary myoblast using TRIzol reagent (Invitrogene, 15596018). cDNA was synthesized from 2 µg of RNA using M-MLV reverse transcriptase (Promega, M170B). qRT-PCR analysis was performed with a QuantiFast SYBR Green PCR Kit 2000 containing cDNA, primers, and SYBR Green PCR master mix (Qiagen, 204156) and data was acquired under condition of two-step cycling, such as denaturation (95°C, 10 s) and combined annealing/extension (58°C, 30 s), for 45 cycles using a StepOnePlus real-time PCR system (Applied Biosystems, Waltham, MA, USA). GAPDH was used as an internal control. Primer sequences are shown in Table S1.

**Western blot analysis.** Protein from TA and quadriceps muscles was prepared with RIPA lysis buffer (Biosesang, Kyunggi, South Korea) and protein concentrations were determined using bicinchoninic acid protein assay (Thermo Fisher Scientific, Rockford, IL, USA). Equal amounts of proteins were separated by 10% of SDS-PAGE and blotted to a polyvinylidene difluoride membrane (Bio-Rad Laboratories, 1620177). To determine the relative levels of protein expression, the intensities of bands in all the blots were measured using densitometric program Image J and the relative band density was calculated by dividing the intensity of each protein band by the intensity of total form or β-actin band.

**5-ethynyl-2'-deoxyuridine (EdU) proliferation assays.** To determine the proliferative capacity of the cells, we used a Click-iT reaction kit (Invitrogen C10640) as per manufacturer’s guidelines using a fluorescence Alexa Fluor 555 to detect the incorporated EdU. In cultured primary myoblast grown on chamber slides (Sigma C7182) coated in matrigel (Corning, 354277), EdU was added to a concentration of 10 mM for 45 min and detected via Click-iT Kit following IF procedures.

**Four limb-hanging test**. In vivo muscle strength was assessed at 9 weeks after DMM surgery and in accordance with a previously reported method^7^. We slightly modified this method for our experimental purpose. Briefly, each mouse was placed on a wire grid mesh surface and it grasps it with the four paws. Then invert the grid so that the mouse is hanging and start a timer. Because DMM surgery was conducted on the right knee joint, the test was stopped when, the right hind paw or a mouse falls off the wire. A fixed hanging limit is used for 60 sec. The test was performed three times, with 15-min intervals.

**Measurement of pain-related behaviors in mice.** Mechanical allodynia was assessed every 2 weeks using von Frey filaments, in accordance with a previously reported method^8^. Briefly, each mouse was placed in a dark glass on a wire mesh surface and allowed to adapt to the testing environment for 5 min. Multiple von Frey filaments with ascending gauges of stiffness (North Coast Medical, Morgan Hill, CA, USA) were then perpendicularly applied to the mouse’s hind paw until the filament bent. The 0.4-g filament was applied five times as the initial force. If the rate of a response, such as a brisk withdrawal or paw flinching, was < 50% (0–2 of 5 applications), the next thicker filament was tested. If the withdrawal response rate was > 50%, the test was stopped and the force of the last von Frey filament was regarded as the mechanical withdrawal threshold. The test was repeated twice for each mouse. Additionally, the withdrawal threshold in response to direct pressure on the medial part of the left knee was measured every 2 weeks using a pressure application measurement device (Ugo Basile, France, PAM-38500) as previously described with some modifications^9^. While a mouse was lightly but securely held, the sensor tip (5-mm diameter) of an applied force gauge was placed on the mouse’s knee joint. An increasingly forceful squeeze was then slowly applied across the joint at a rate of 50 g/s, with a maximum test duration of 30 s. The test was stopped when the mouse showed any behavioral signs of discomfort or distress, such as wriggling. The peak gram force (gf) applied immediately prior to limb withdrawal was recorded as the base unit and designated as the limb withdrawal threshold. A cut-off force of 500 g was used to prevent trauma to the joint. The test was performed three times, with 10-min intervals, and the mean value was recorded as the nociceptive threshold.

**Determinantion of sample size.** Sample size for each experiment was determined using G power program (<https://clincalc.com/stats/samplesize.aspx>). For von Frey test we hypothesized mean Von Frey scores in male mice to be 1.9 (SD 0.5) for the sham group vs. 1.1 (SD 0.25) in the DMM group. Given 80% power and an alpha level of 0.05, the power calculation suggested 6 male mice per group. In the same manner as Von Frey score, mean pressure scores were hypothesized to be 450 (SD 33.75) vs. 382 (SD 21.2) for male sham and DMM group. Given 80% power and an alpha level of 0.05, the power calculation suggested 4 male mice per group. Given concerns about the ability of the animals to complete the experiments we increased the sample sizes to 6 and 9.

Table S1. Primer sequences for mouse genes analyzed by qRT-PCR

| **Gene** | **Primer sequence (5’-3’)** |
| --- | --- |
| GAPDH | Forward-CAT GGC CTT CCG TGT TCC |
|  | Reverse-GCG GCA CGT CAG ATC CA |
| FBXO32 | Forward-GTG CAG AGA GTC GGC AAG TCT |
|  | Reverse-CCA TCC GAT ACA CCC ACA TG |
| TRIM63 | Forward-TTC ATC GAG GCT CTG ATC CTC CA |
|  | Reverse-AGT CCA TGT TCT CAA AGC CTT GCT |
| MCP-1 | Forward-GGT CTG TGC TGA CCC CAA GA |
|  | Reverse-TTT GGT TCC GAT CCA GGT TTT |
| IL-1b | Forward-GCA CCT TCT TTT CCT TCA TCT TTG |
|  | Reverse-GTT GTT CAT CTC GGA GCC TGT |
| IL-6 | Forward-TTC ACA GAG GAT ACC ACT CCC AAC A |
|  | Reverse-CTG CAA GTG CAT CAT CGT TGT TCA T |
| MyoD | Forward-CGG CTC TCT CTG CTC CTT TG |
|  | Reverse-GAG TCG AAA CAC GGG TCA TCA |
| Myogenin | Forward-CGG CTG CCT AAA GTG GAG AT |
|  | Reverse-AGG CCT GTA GGC GCT CAA T |
| Desmin | Forward-ACA TCC GGG CTC AGT ATG AGA |
|  | Reverse-CTT GTA CCA TTC TTC AGC CTC AGA |
| ACAN | Forward-GGA CAG GCT GGC TGA AGA |
|  | Reverse-GAG ACC CCT GGG AAG GAA |
| Col2a1 | Forward-GTG TGT GTG ACA CTG GGA ATG TC |
|  | Reverse-GGT TGA GGC AGT CTG GGT CTT |
| Sox9 | Forward-CGG CTC CAG CAA GAA CAA G |
|  | Reverse-GCG CCC ACA CCA TGA AG |


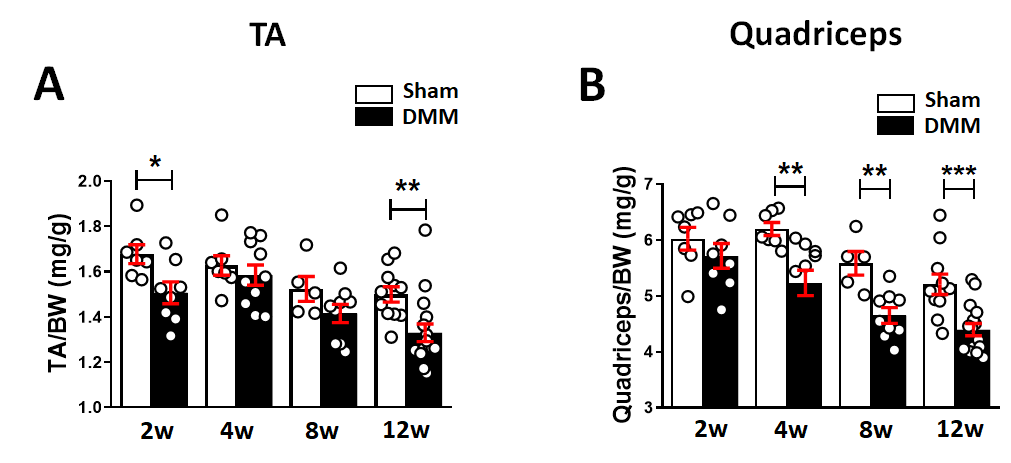


Supplementary Figure 1. Muscle mass is reduced during the progression of OA. **A, B**, Relative muscle mass (mg per g body weight) of the tibialis anterior (TA) (A) and quadriceps (B) muscles at 2, 4, 8, and 12 weeks after DMM or sham surgery (n = 7–16). Data are shown as means ± SEMs. *P < 0.05, **P < 0.01, ***P < 0.001 according to unpaired two-tailed *t*-tests.


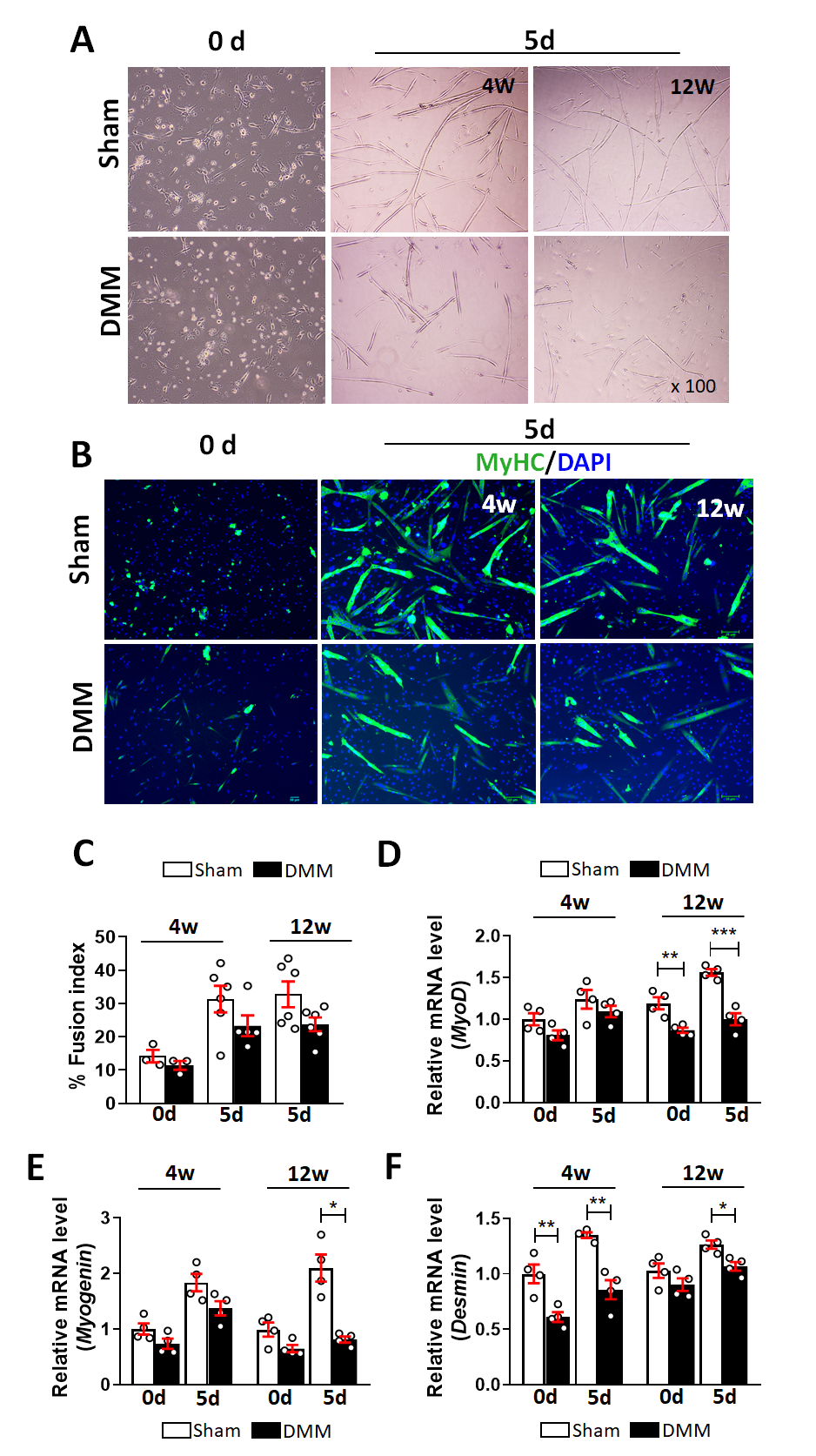


Supplementary Figure 2. The myogenic commitment of myoblast cells is reduced during OA development. Primary myoblast cells were isolated from pooled TA and quadriceps muscles of sham or 4- or 12-week postoperative DMM mice (n = 5 per group), then allowed to differentiate for 5 days. **A**, Representative bright-field microscopy images (100× magnification) of cultured primary myoblast cells from the TA and quadriceps muscles of sham-operated or 4- or 12-week postoperative DMM mice. **B**, Representative immunofluorescence images of cultured myoblast cells stained for MyHC (green) and labeled with 4′,6-diamidino-2-phenylindole (DAPI; blue). Scale bars = 50 μm. **C**, Fusion index after 5 days of differentiation, calculated as the percentage of total nuclei in cells containing ≥ 3 nuclei. At least five images per cell were examined. **D, E, and F**, Relative mRNA levels of three markers of myogenic differentiation (MyoD (D), myogenin (E), and desmin (F)) in primary myoblast cells on day 0 and after 5 days of differentiation. Data are normalized to the expression of GAPDH mRNA and shown as means ± SEMs of duplicate experiments. *P < 0.05, **P < 0.01, ***P < 0.001 according to two-tailed Mann–Whitney U test (C) or unpaired two-tailed *t*-tests (D, E, F).


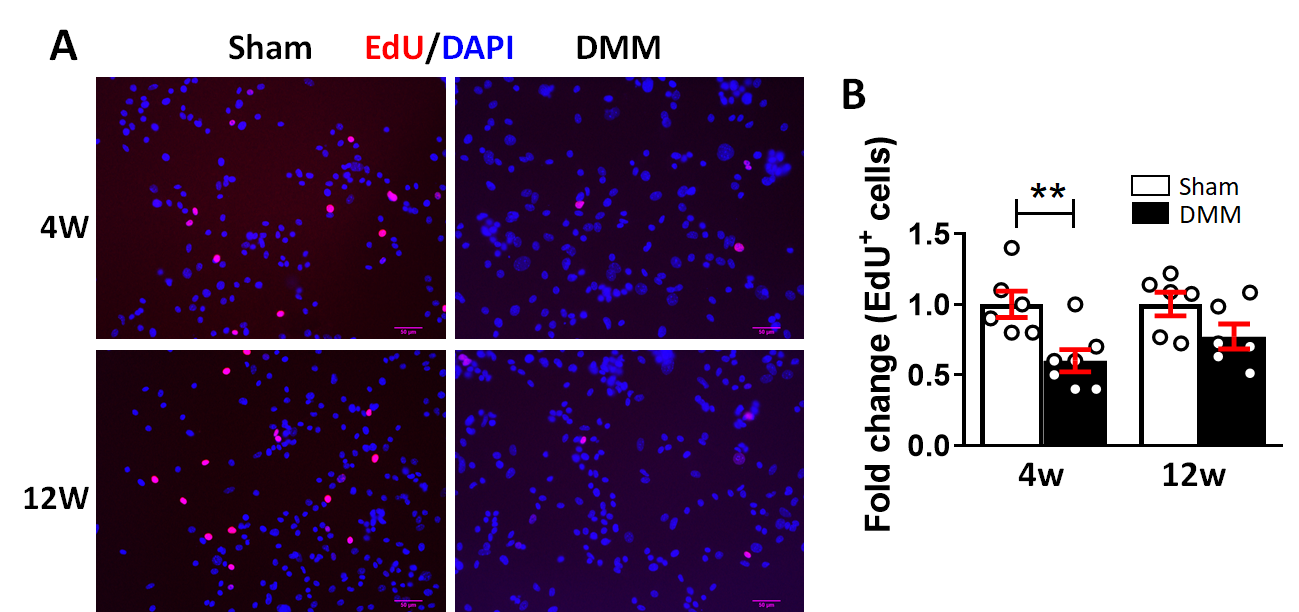


Supplementary Figure 3. The proliferation of myoblast cells decreased during OA development. Primary myoblast cells were isolated from pooled TA and quadriceps muscles of sham-operated or 4- or 12-week postoperative DMM mice (n = 5 per group). **A**, EdU immunofluorescence in primary cultured myoblasts. EdU (red), DAPI (blue), and merged (pink). Scale bars = 50 μm. **B**, Fold increase in EdU-positive myoblasts (%). At least six fields per cell were examined. Data are shown as means ± SEMs. **P < 0.01 according to unpaired two-tailed *t*-tests.


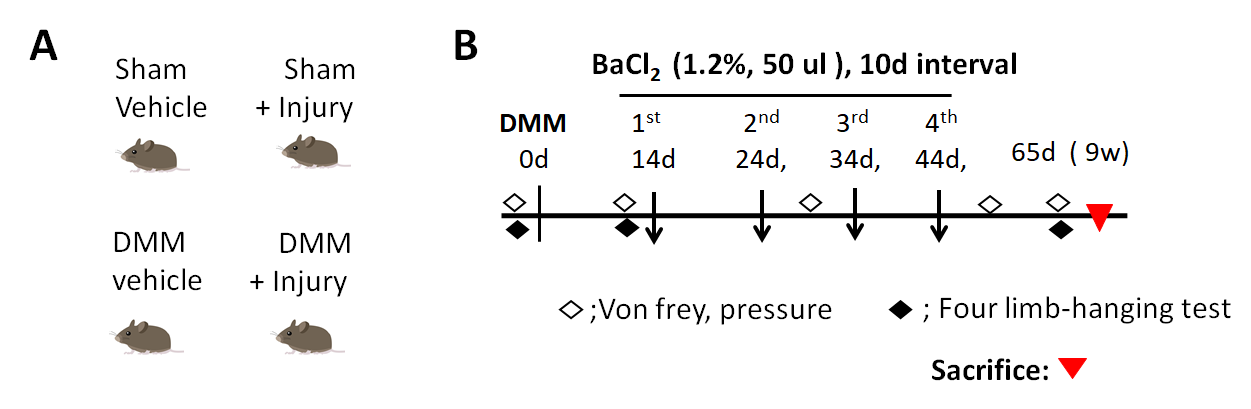


Supplementary Figure 4. Experimental design for the induction of muscle weakness by multiple rounds of BaCl_2_ injection into the quadriceps muscle. **A**, Ten-week-old male C57BL/6 mice were divided into four experimental groups: sham vehicle, sham+injury, DMM vehicle, and DMM+injury. **B**, Periarticular muscle weakness was established by injecting BaCl_2_ into quadriceps muscles four times at 10-day intervals (arrows), without allowing complete regeneration between injections. Quadriceps muscles and knee joints were collected 3 weeks after the last injection and 9 weeks after DMM. Sham or DMM vehicle mice were injected with phosphate-buffered saline. Pain was measured using von Frey filaments and a pressure algometer at the indicated time points. Hind limb strength was measured in a four limb-hanging test.


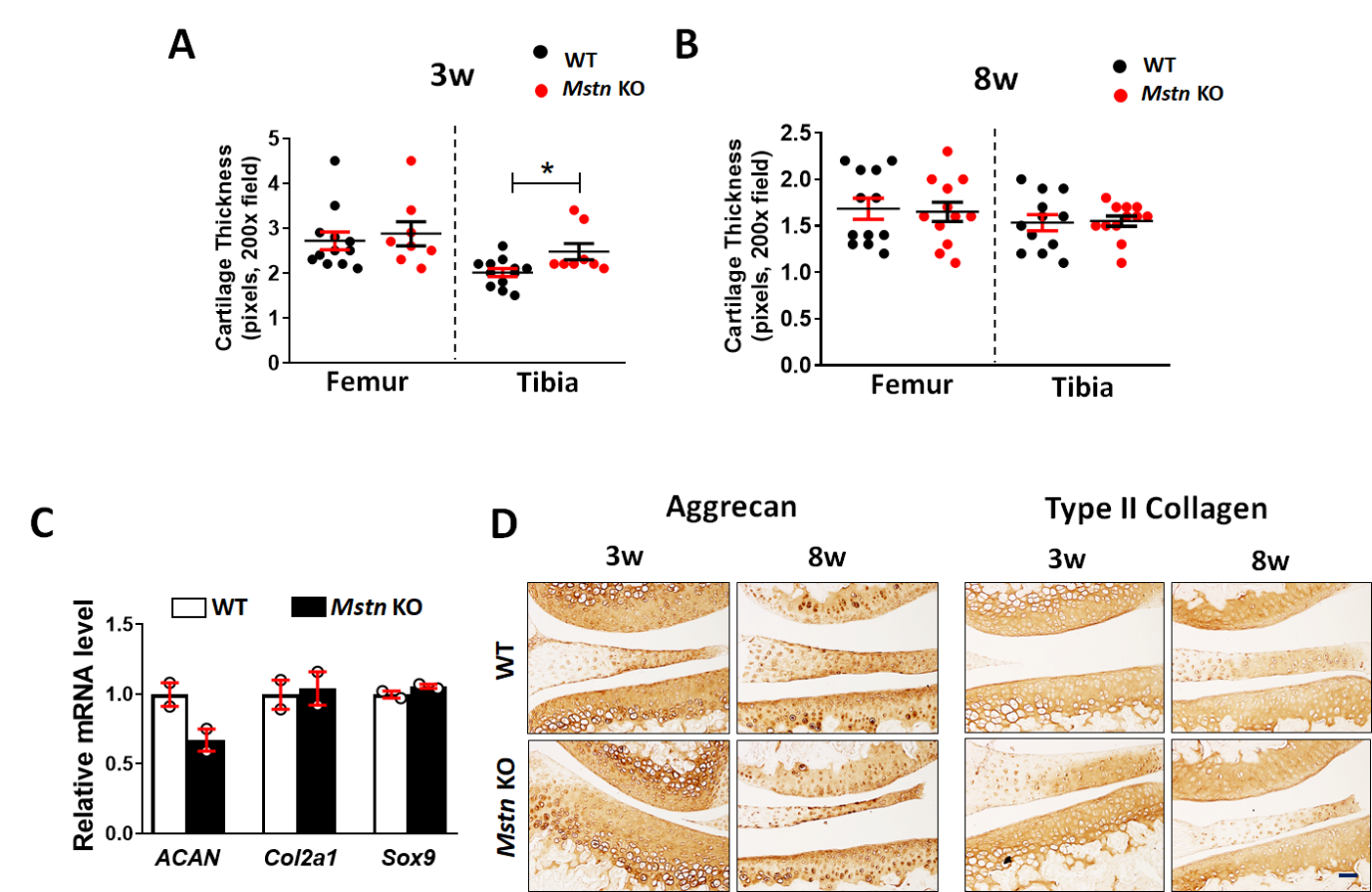


Supplementary Figure 5. **A and B**, Quantification of articular cartilage thickness in the femoral condyle or tibial plateau in WT and Mstn KO mice at 3 (n = 8–12) and 8 (n = 12) weeks of age. Measurements from the subchondral bone to the articular cartilage surface were made at three points across the tibial plateau and femoral condyle of the medial compartment, and the values were averaged. **C**, Relative mRNA levels of ACAN, Col2a1, and Sox9 from pooled tibial and femoral cartilage in 8-week-old WT (n = 10) and Mstn KO (n = 10) mice were analyzed using real-time qRT-PCR. Data are normalized to the expression of GAPDH mRNA and are shown as means ± SEMs of duplicate experiments. **D,** Representative image of immunohistochemical staining for aggrecan and Type II collagen in knee sections from 3- and 8-week-old WT and Mstn KO mice. Scale bars = 50 μm. Data are shown as means ± SEMs. *P < 0.05 according to two-tailed Mann–Whitney U test in (A, femur, tibia), (B, femur) and (C), and according to unpaired two-tailed *t*-tests in (B, tibia).


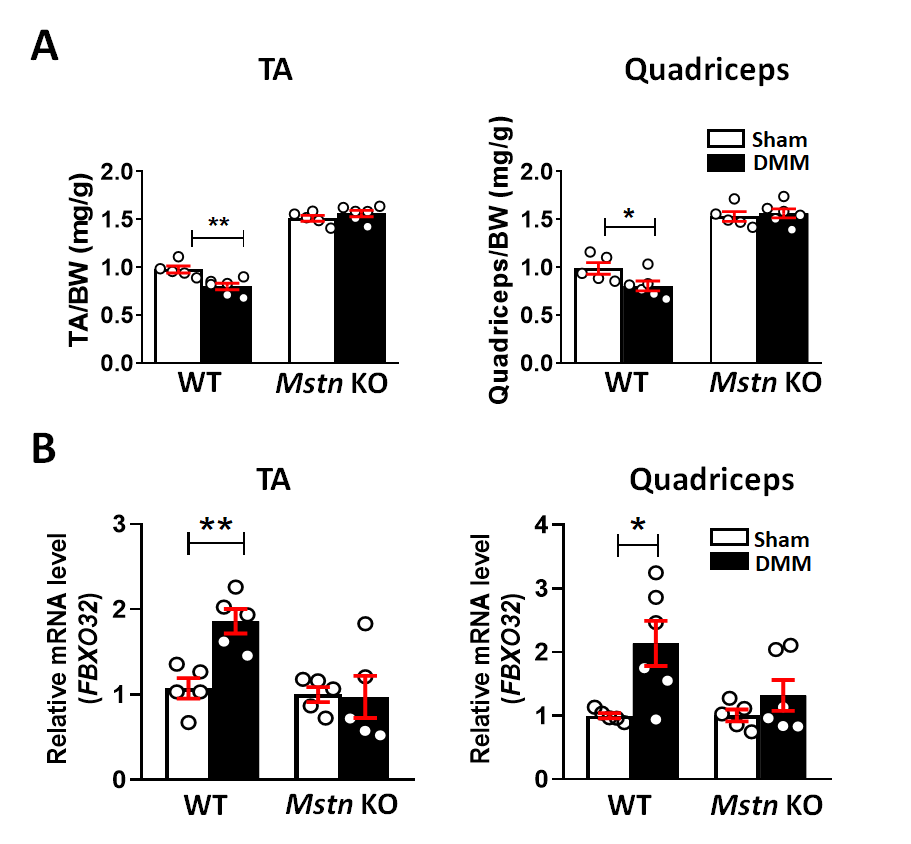


Supplementary Figure 6. Muscle mass was not reduced after induction of OA in Mstn KO mice. **A,** Relative muscle mass (mg per g body weight) of the tibialis anterior (TA) and quadriceps muscles at12 weeks after DMM or sham surgery (n = 5–6). **B,** Expression levels of atrogin-1 mRNA in the TA and quadriceps muscles of the sham-operated (n = 5) and DMM (n = 5-6) groups, as determined by real-time qRT-PCR. Data are shown as means ± SEMs. *P < 0.05, **P < 0.01 according to unpaired two-tailed *t*-tests.

**Supplementary Methods References**

1 Glasson, S. S., Blanchet, T. J. & Morris, E. A. The surgical destabilization of the medial meniscus (DMM) model of osteoarthritis in the 129/SvEv mouse. *Osteoarthritis Cartilage* **15**, 1061-1069, doi:10.1016/j.joca.2007.03.006 (2007).

2 Glasson, S. S., Chambers, M. G., Van Den Berg, W. B. & Little, C. B. The OARSI histopathology initiative - recommendations for histological assessments of osteoarthritis in the mouse. *Osteoarthritis Cartilage* **18 Suppl 3**, S17-23, doi:10.1016/j.joca.2010.05.025 (2010).

3 Krenn, V. *et al.* Synovitis score: discrimination between chronic low-grade and high-grade synovitis. *Histopathology* **49**, 358-364, doi:10.1111/j.1365-2559.2006.02508.x (2006).

4 Furman, B. D. *et al.* Joint degeneration following closed intraarticular fracture in the mouse knee: a model of posttraumatic arthritis. *J Orthop Res* **25**, 578-592, doi:10.1002/jor.20331 (2007).

5 Kamekura, S. *et al.* Osteoarthritis development in novel experimental mouse models induced by knee joint instability. *Osteoarthritis Cartilage* **13**, 632-641, doi:10.1016/j.joca.2005.03.004 (2005).

6 Springer, M. L., Rando, T. A. & Blau, H. M. Gene delivery to muscle. *Curr Protoc Hum Genet* **Chapter 13**, Unit13 14, doi:10.1002/0471142905.hg1304s31 (2002).

7 Bonetto, A., Andersson, D. C. & Waning, D. L. Assessment of muscle mass and strength in mice. *Bonekey Rep* **4**, 732, doi:10.1038/bonekey.2015.101 (2015).

8 Hwang, H. S., Park, I. Y., Hong, J. I., Kim, J. R. & Kim, H. A. Comparison of joint degeneration and pain in male and female mice in DMM model of osteoarthritis. *Osteoarthritis Cartilage* **29**, 728-738, doi:10.1016/j.joca.2021.02.007 (2021).

9 Sun, Q. *et al.* Parathyroid hormone attenuates osteoarthritis pain by remodeling subchondral bone in mice. *Elife* **10**, doi:10.7554/eLife.66532 (2021).

Supplementary data for the Figure 5H, Original western blot images of atrogin-1 and β-actin

**Atrogin-1 (42 kDa)**


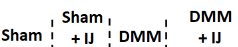

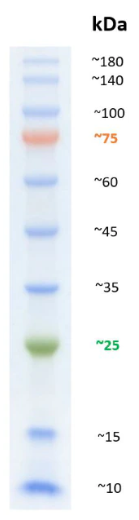

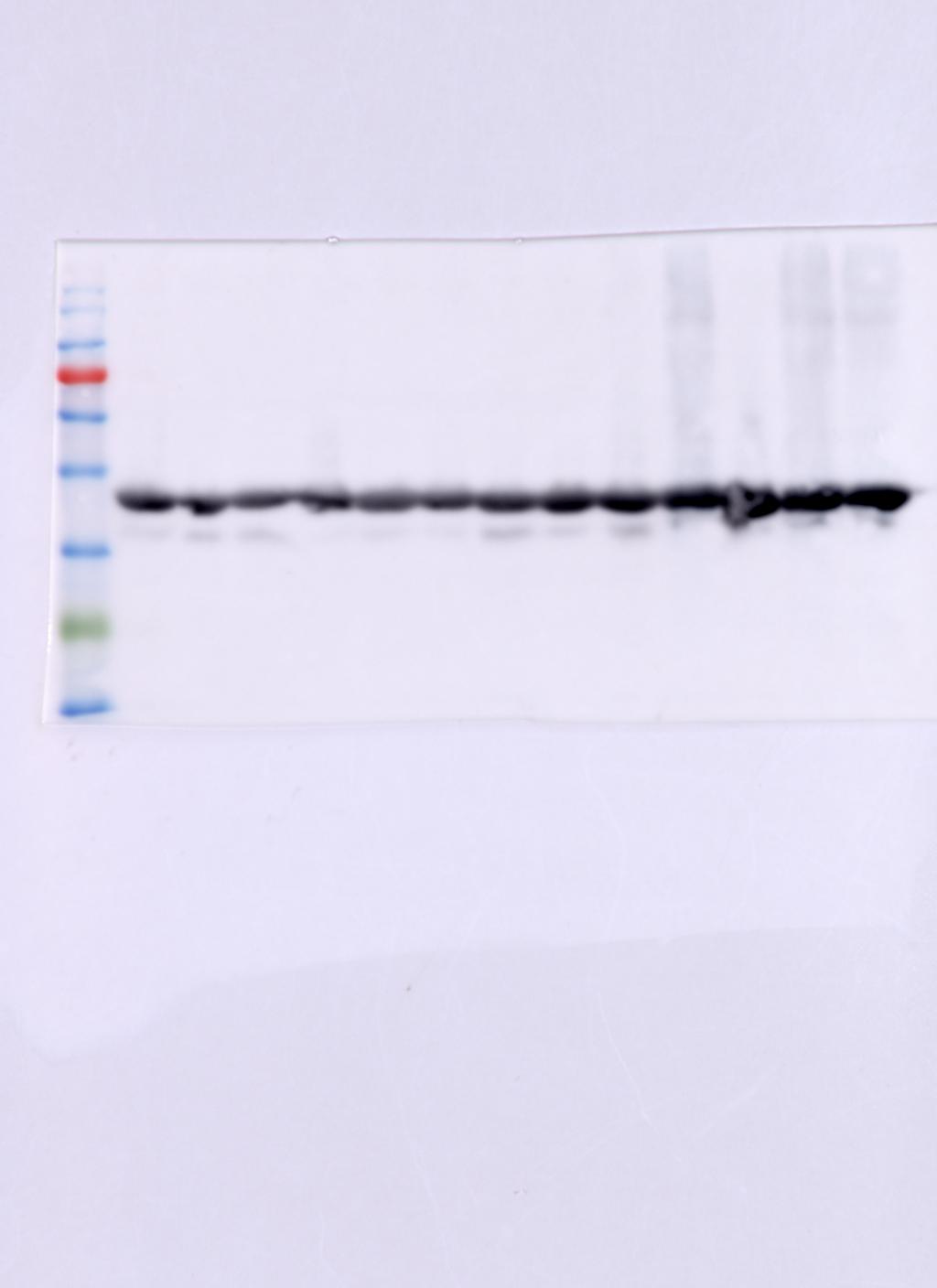
Protein size marker

kDa

42 kDa

45

30

60

75

**beta-actin (42 kDa)**


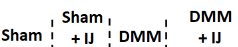

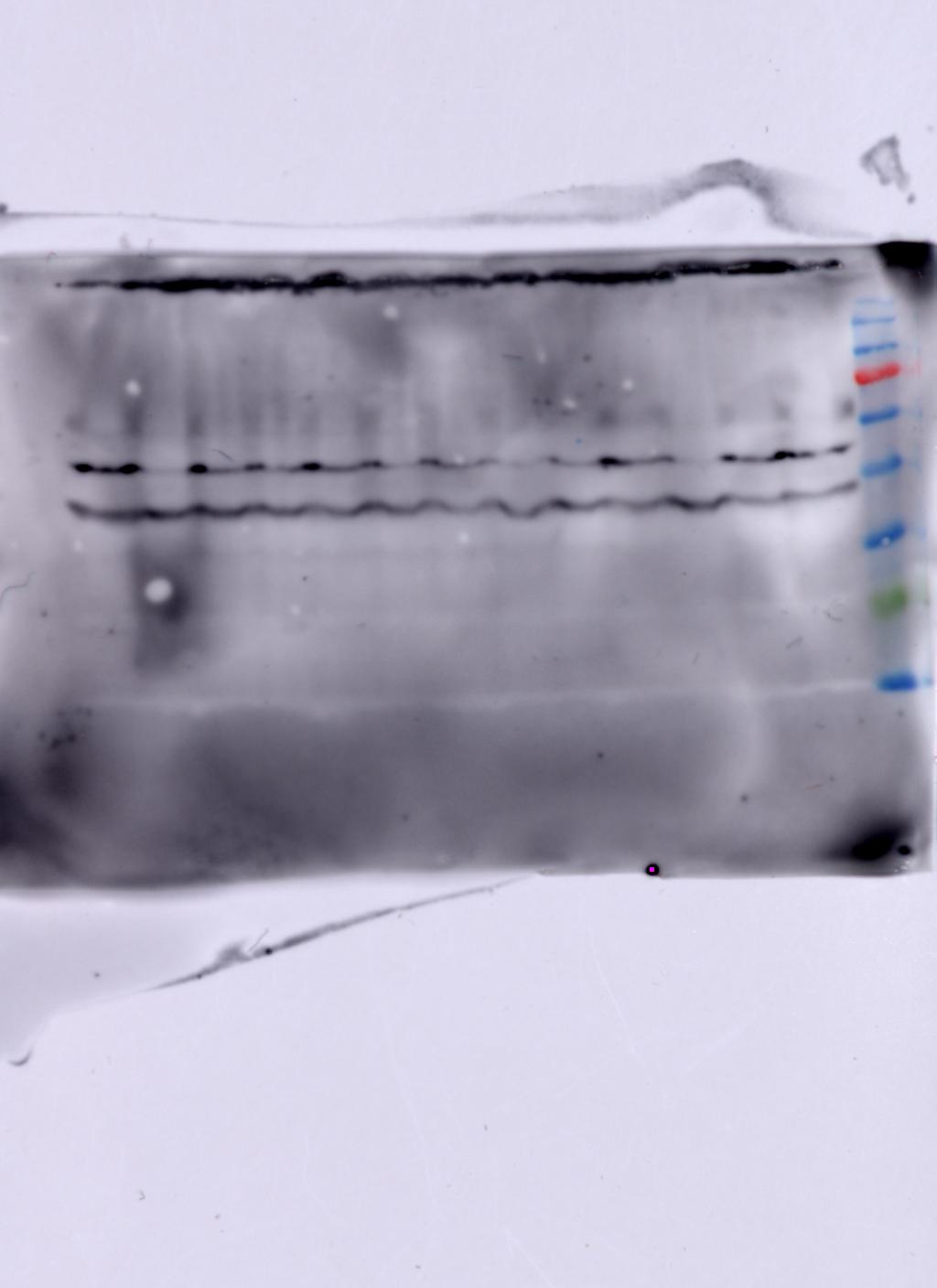


42 kDa

Supplementary data 1. Display of original blots for atrogin-1 and β-actin Western blot analysis ( for Figure 5H)
